# Impact of slow breathing on the blood pressure and subarachnoid space width oscillations in humans

**DOI:** 10.1038/s41598-019-42552-9

**Published:** 2019-04-17

**Authors:** Magdalena K. Nuckowska, Marcin Gruszecki, Jacek Kot, Jacek Wolf, Wojciech Guminski, Andrzej F. Frydrychowski, Jerzy Wtorek, Krzysztof Narkiewicz, Pawel J. Winklewski

**Affiliations:** 1 Department of Human Physiology, Faculty of Health Sciences, Medical University of Gdansk, Gdansk, Poland; 2 Department of Radiology Informatics and Statistics, Faculty of Health Sciences, Medical University of Gdansk, Gdansk, Poland; 3 National Centre for Hyperbaric Medicine, Institute of Maritime and Tropical Medicine, Faculty of Health Sciences, Medical University of Gdansk, Gdynia, Poland; 4 Department of Hypertension and Diabetology, Faculty of Medicine, Medical University of Gdansk, Gdansk, Poland; 5 Department of Computer Communications, Faculty of Electronics, Telecommunications and Informatics, Gdansk University of Technology, Gdansk, Poland; 6 Department of Biomedical Engineering, Faculty of Electronics, Telecommunications and Informatics, Gdansk University of Technology, Gdansk, Poland; 7 Department of Clinical Anatomy and Physiology, Faculty of Health Sciences, Pomeranian University of Slupsk, Slupsk, Poland

**Keywords:** Blood flow, Hypertension

## Abstract

The aim of the study was to assess cardiac and respiratory blood pressure (BP) and subarachnoid space (SAS) width oscillations during the resting state for slow and fast breathing and breathing against inspiratory resistance. Experiments were performed on a group of 20 healthy volunteers (8 males and 12 females; age 25.3 ± 7.9 years; BMI = 22.1 ± 3.2 kg/m^2^). BP and heart rate (HR) were measured using continuous finger-pulse photoplethysmography. SAS signals were recorded using an SAS monitor. Oxyhaemoglobin saturation (SaO_2_) and end-tidal CO_2_ (EtCO_2_) were measured using a medical monitoring system. Procedure 1 consisted of breathing spontaneously and at controlled rates of 6 breaths/minute and 6 breaths/minute with inspiratory resistance for 10 minutes. Procedure 2 consisted of breathing spontaneously and at controlled rates of 6, 12 and 18 breaths/minute for 5 minutes. Wavelet analysis with the Morlet mother wavelet was applied for delineation of BP and SAS signals cardiac and respiratory components. Slow breathing diminishes amplitude of cardiac BP and SAS oscillations. The overall increase in BP and SAS oscillations during slow breathing is driven by the respiratory component. Drop in cardiac component of BP amplitude evoked by slow-breathing may be perceived as a cardiovascular protective mechanism to avoid target organ damage. Further studies are warranted to assess long-term effects of slow breathing.

## Introduction

Slow breathing practices have been practiced for thousands of years amongst Eastern cultures due to their perceived health benefits^1^. Yogic breathing (pranayama) represents an example of such ancient practice of controlled breathing, often performed in conjunction with meditation or yoga^2,3^. Significantly, a number of studies based on modern experimental and computational approaches have confirmed that ancient techniques with controlled respiration may exert a beneficial impact on overall well-being. For instance, resonant breathing (6 breaths per minute) results in improvements in cardiovascular functions such as blood flow to internal organs and sensitivity of the sympathetic component of the baroreflex and ventricular elastance^4–6^. Various yoga techniques based on slow breathing were also reported to improve cognitive functions among their practitioners^7^. Several studies have demonstrated a reduction in acute mean arterial pressure (MAP) during controlled slow respiration^5,8,9^.

Controlled slow breathing, particularly at 6 breaths per minute, is associated with augmented BP fluctuations of respiratory origin, as compared to the BP oscillations observed during spontaneous breathing^4,8,10^. However, it remains unclear how controlled slow breathing affects BP oscillations of cardiac origin. Delineation of the cardiac and respiratory components may help to better understand those BP-related mechanisms that are implicated in cardiovascular disease. Importantly, the calculating wavelet amplitude ensures the identification of cardiac and respiratory components during provocative tests.

Several studies have suggested that one of the fundamental roles of cerebrospinal fluid (CSF) is buffering detrimental cerebral blood flow pulsatility^11^; however, the regulatory mechanisms underlying CSF motion have yet to be unveiled. Evidently, back-and-forth periodic CSF motion is secondary to cardiac and respiratory cycles (reviewed recently by^12^). Nevertheless, the precise description of these two physiological processes is difficult due to the technical shortcomings of magnetic resonance imaging (MRI). Apart from inadequate resolution, the imaging of CSF motion during the cardiac and respiratory cycles requires different MRI sequences, a fact which precludes simultaneous measurements of these two components^13^.

Recently, we proposed using subarachnoid space (SAS) width pulsatility as a surrogate for CSF pulsatile flow^12^. Periodic oscillations of SAS width can be measured non-invasively in humans with a near-infrared transillumination/backscattering sounding (NIR-T/BSS) method. In short, the main assumption for NIR-T/BSS technique is that translucent CSF in SAS acts as a propagation duct for infrared radiation (a technique resembling optical fibers engineering)^14,15^. This allows for measurement of the SAS width to estimate changes in CSF volume^14–18^. NIR-T/BSS has been validated against MRI, showing comparable SAS width alterations induced by shifts in body position. Regression analysis of data derived from both methods shows that SAS width alterations observed in supine vs. abdominal-lying positions yield high interdependence between both methods (R  =  0.81, P < 0.001)^19^.

Importantly, in accordance with the Nyquist theorem, the NIR-T/BSS high sampling frequency allows for signal analysis up to 35 Hz. Thus, as no other available technique does, the NIR-T/BSS accurately identifies rapid SAS width changes resulting from systole-diastole fluctuations in the cerebral blood volume^20^. Recent analyses have shown that the power spectrum density levels of SAS oscillations are characterized by detectable peaks at both cardiac and respiratory frequencies^21–23^. Taken together, NIR-T/BSS is capable of synchronized assessment of instant cardiac and respiratory influences on SAS width, an exclusive feature of this technique^21–23^.

BP and SAS fluctuations are further augmented by inspiratory resistance due to more negative intrathoracic pressures (reviewed by^1^). In particular, more negative intrathoracic pressures lead to substantial increase in respiratory CSF shifts between cranial compartment and the cervical spinal canal (reviewed by^24^). Thus, increased respiratory resistance should exacerbate the effects of slow breathing on the investigated variables. The aim of the present study was to delineate cardiac- and respiratory-generated BP and SAS oscillations during the resting state, slow and fast breathing and breathing against respiratory resistance. We hypothesized that induced slow breathing will augment amplitudes of BP and SAS respiratory oscillations while at the same time it may diminish amplitudes of BP and SAS cardiac oscillations.

## Results

### Procedure 1

Non-parametric Friedman test showed that there was a significant differences (p < 0.05) for all measured signals (excluding EtCO_2_) during all stages of procedure 1. We also showed (Table 1) which stages of procedure are significantly different from each other (St. 1 and 3 means that stage 1 is significantly different from stage 3).

**Table 1:** Procedure 1.

	Baseline (1)	6 breaths/min (2)	6 breaths/min + Resistance (3)	Recovery (4)
HR [beats/min]	76.2 ± 9.6	76.8 ± 10.8	78.1 ± 9.6	76.2 ± 16.2
**Friedman Test**	**p** = **0.008**	**Post hoc**	**St. 1 and 3**	
DBP [mmHG]	63.58 ± 10.19	58.67 ± 12.88	57.66 ± 13.72	55.98 ± 13.97
**Friedman Test**	**p** = **0.003**	**Post hoc**	**St. 1 and 2, St. 1 and 3**	**St. 1 and 4**
SBP [mmHG]	116.91 ± 9.16	111.74 ± 13.79	112.92 ± 17.88	107.18 ± 15.96
**Friedman Test**	**p** = **0.01**	**Post hoc**	**St. 1 and 4**	
MAP [mmHG]	81.89 ± 8.65	77.41 ± 11.89	77.84 ± 13.13	75.01 ± 12.66
**Friedman Test**	**p** = **0.001**	**Post hoc**	**St. 1 and 2, St. 1 and 4**	
SAS_LEFT_ [AU]	957 ± 537	969 ± 462	1323 ± 714	1077 ± 639
**Friedman Test**	**p** = **0.03**	**Post hoc**	**St. 1 and 3**	
SAS_RIGHT_ [AU]	855 ± 273	1053 ± 471	1392 ± 738	1071 ± 663
**Friedman Test**	**p** = **0.002**	**Post hoc**	**St. 1 and 3, St. 3 and 4**	
SaO_2_	98.17 ± 0.11	99.05 ± 0.39	99.45 ± 0.46	98.83 ± 0.28
**Friedman Test**	**p** = **0.02**	**Post hoc**	**St. 1 and 2, St. 1 and 3**	
EtCO_2_	36.77 ± 1.11	35.63 ± 1.68	36.13 ± 1.23	36.93 ± 1.44
**Friedman Test**	**p** = **0.1**	**Post hoc**	—	

### Procedure 2

Non-parametric Friedman test showed that there was a significant differences (p < 0.05) for four variables: HR, DBP, MAP and SaO_2_. We also showed which stages of procedure are significantly different from each other (Table 2).

**Table 2:** Procedure 2.

	Baseline (1)	6 breaths/min (2)	12 breaths/min (3)	18 breaths/min (4)	Recovery (5)
HR [beat/min]	74.4 ± 6.6	79.8 ± 21	85.2 ± 19.8	86.4 ± 20.4	81.6 ± 26.4
**Friedman Test**	**p** = **0.007**	**Post hoc**	**St. 1 and 3, St. 1 and 4**		
DBP [mmHG]	71.99 ± 7.74	69.32 ± 9.05	66.66 ± 9.15	65.07 ± 10.59	66.57 ± 10.81
**Friedman Test**	**p** = **0.0005**	**Post hoc**	**St. 1 and 3, St. 1 and 4, St. 1 and 5**		
SBP [mmHG]	113.91 ± 15.67	113.16 ± 15.13	109.83 ± 17.94	107.11 ± 20.97	109.37 ± 21.45
**Friedman Test**	**p** = **0.06**	**Post hoc**	**---------------------**		
MAP [mmHG]	87.15 ± 7.94	84.69 ± 8.91	81.67 ± 9.91	79.48 ± 12.33	81.23 ± 12.72
**Friedman Test**	**p** = **0.002**	**Post hoc**	**St. 1 and 4**		
SAS_LEFT_ [AU]	864 ± 507	1059 ± 789	918 ± 558	858 ± 555	1053 ± 708
**Friedman Test**	**p** = **0.83**	**Post hoc**	**---------------------**		
SAS_RIGHT_ [AU]	801 ± 492	1113 ± 483^NS^	978 ± 555	957 ± 702	1032 ± 717
**Friedman Test**	**p** = **0.21**	**Post hoc**	**---------------------**		
SaO_2_	98.23 ± 0.09	99.15 ± 0.43	99.32 ± 0.31	99.54 ± 0.62	98.17 ± 0.68
**Friedman Test**	**p** = **0.006**	**Post hoc**	**St. 1 and 2, St. 1 and 3, St. 1 and 4**		
EtCO_2_	36.63 ± 1.09	35.83 ± 1.73	36.21 ± 1.15	35.93 ± 1.87	36.23 ± 1.54
**Friedman Test**	**p** = **0.16**	**Post hoc**	**---------------------**		

**Figure 1:**
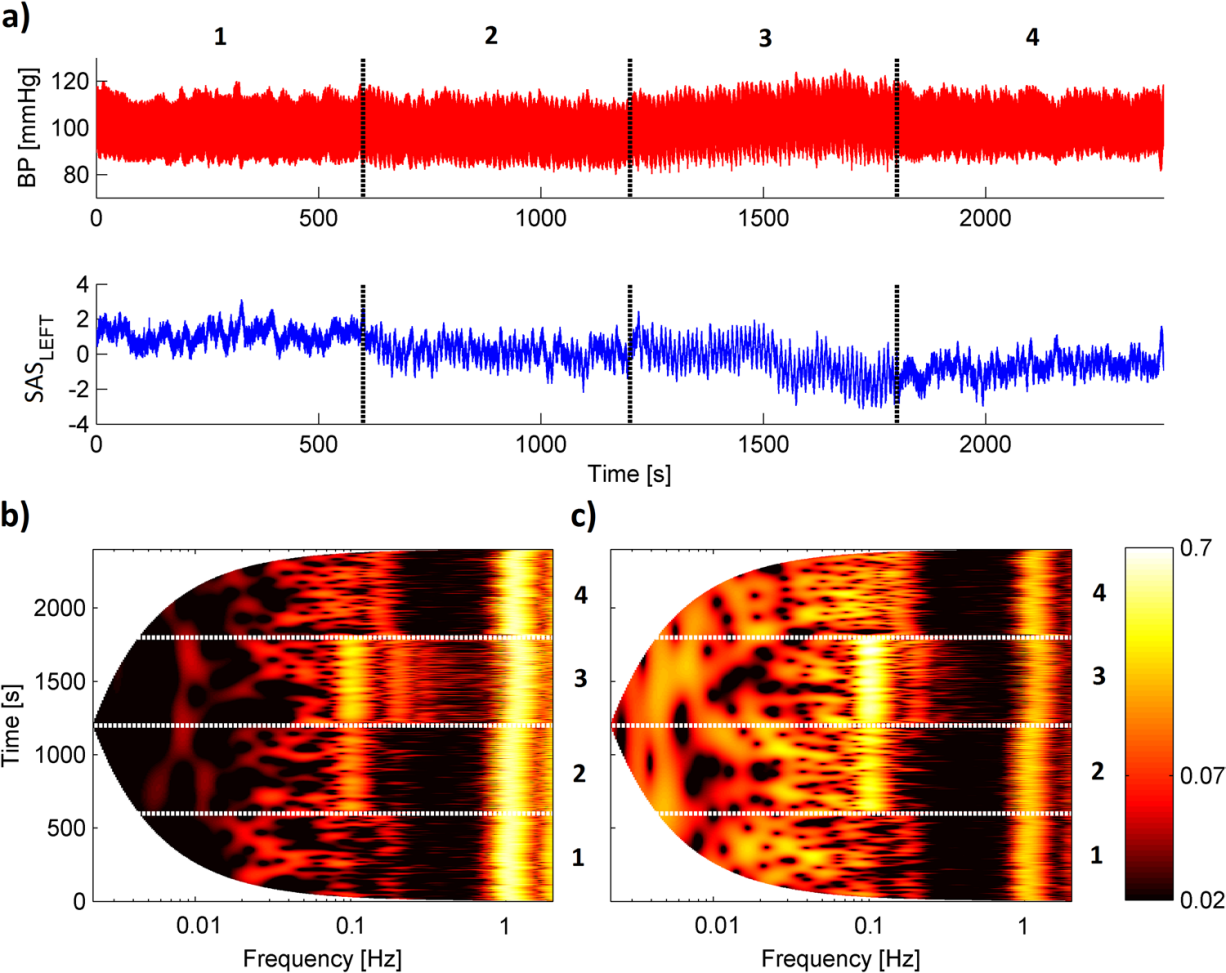
(**a**) Simultaneous recordings of (top panel) blood pressure and (bottom panel) SAS width (left hemisphere) signals measured for one subject. (**b,c**) Wavelet transforms of the whole recording for (**b**) blood pressure and (**c**) SAS width signal. Four numbers correspond to different stages of procedure: (1) baseline spontaneous breathing, (2) breathing at controlled rates of 6 breaths/minute, (3) breathing at controlled rates of 6 breaths/minute with inspiratory resistance and (4) recovery spontaneous breathing.

**Figure 2:**
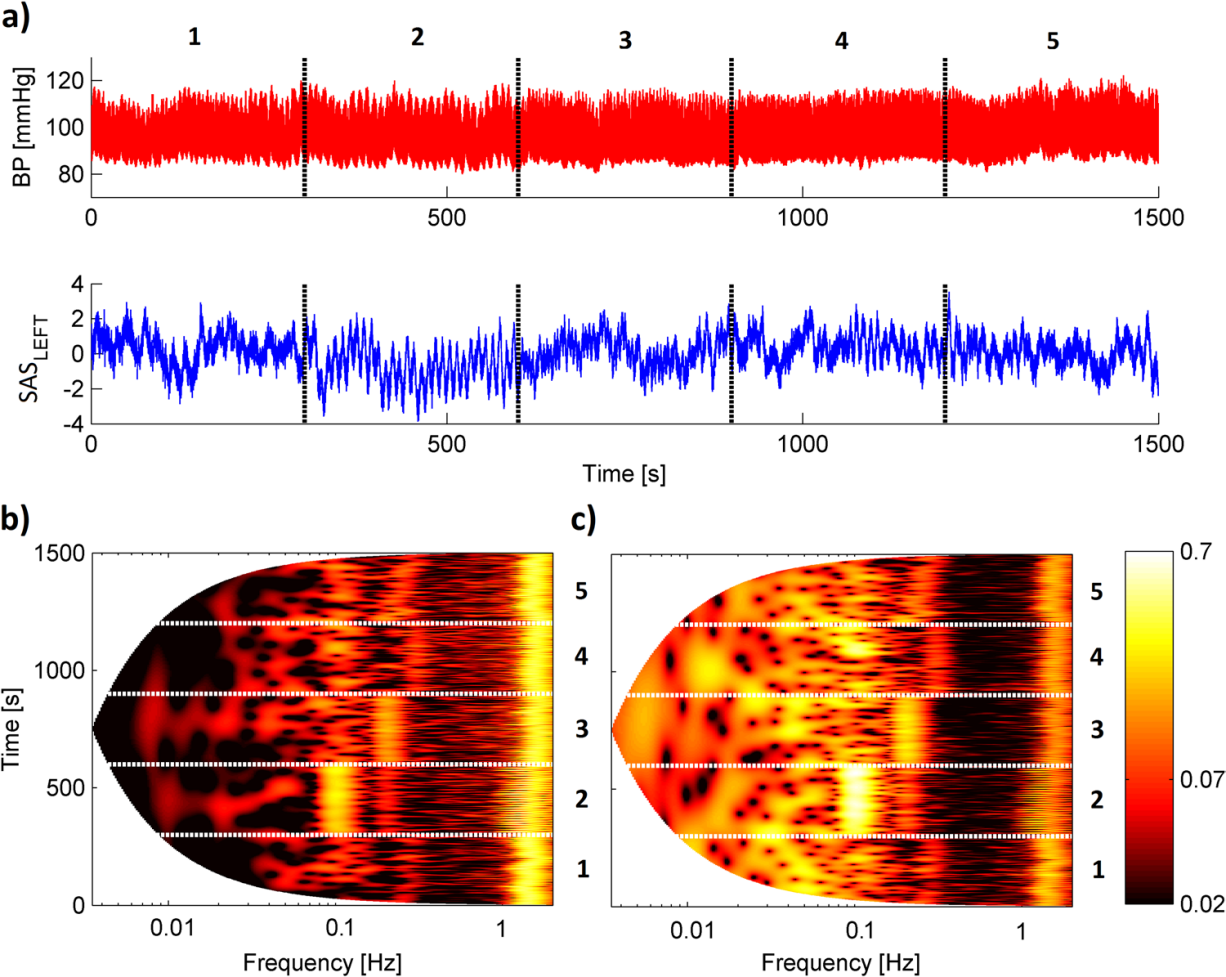
(**a**) Simultaneous recordings of (top panel) blood pressure and (bottom panel) SAS width (left hemisphere) signals measured for one subject. (**b,c**) Wavelet transforms of the whole recording for (**b**) blood pressure and (**c**) SAS width signal. Five numbers correspond to different stages of procedure: (1) baseline spontaneous breathing, (3) breathing at controlled rates of 6 breaths/minute, (3) breathing at controlled rates of 12 breaths/minute, (4) breathing at controlled rates of 18 breaths/minute and (5) recovery spontaneous breathing.

The top panels of Fig. 1(a) (Fig. 2(a)) illustrate the 40-minute (25-minute) segment of procedure 1 (procedure 2). The bottom panels of Figs 1 and 2(b,c) show the result of applying the wavelet transform. Panel b (c) corresponds to the BP (SAS_LEFT_) signal. Wavelet analysis with the Morlet mother wavelet can detect oscillations with logarithmic frequency resolution and follow the variations of their frequencies and amplitude in time^25,26^. The minimal frequency is not the same for procedures 1 and 2 due to the different time of the signals. It is clearly visible that collected signals manifest over a wide frequency range, but in our study we were interested only in two frequency bands which correspond to cardiac and respiration activity. Previous studies revealed that intervals (0.6 – 2 Hz) and (0.145 – 0.6 Hz) are related to the cardiac and respiration functions^25^. We extended the bottom limit of the respiration interval to 0.1 Hz because in both procedures we asked volunteers to take a breath 6 times per minute, which gives us a frequency of 0.1 Hz. For all signals, we observed a similar cardiac component with a frequency of about 1 Hz. Additionally, there are clearly visible respiration components 0.1 Hz (6 breaths per minute), 0.2 Hz (12 breaths per minute) and 0.3 Hz (18 breaths per minute) for the second procedure (see bottom panels of Fig. 2).

**Figure 3:**
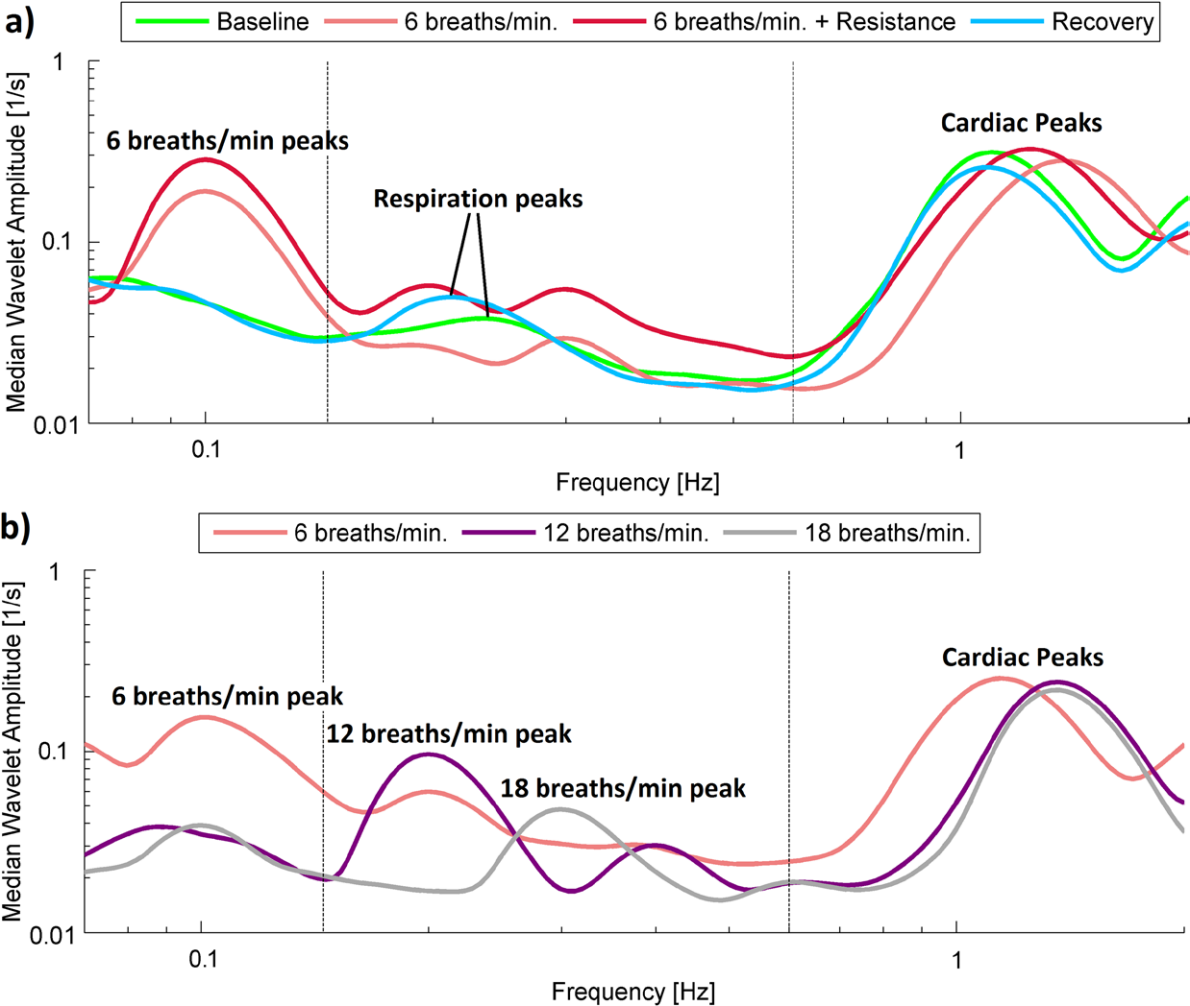
(**a,b**) Median of the time-averaged wavelet transforms of blood pressure signals recorded in all subjects for (**a**) procedure 1 and (**b**) procedure 2. Various line colours correspond to different stages of procedure.

To simplify the comparison of measured signals in terms of their frequency content, we plotted Fig. 3, which illustrates the median of the time-averaged amplitude of wavelet transforms of the time series for procedure 1 (a) and (b). It is clearly visible that there are several peaks in each spectrum, which correspond to the cardiac and respiration activity of our volunteers. The values of the peaks for each volunteer were subjected to statistical analysis to establish more quantitatively the difference between subjects for different stages of the procedures.

**Figure 4:**
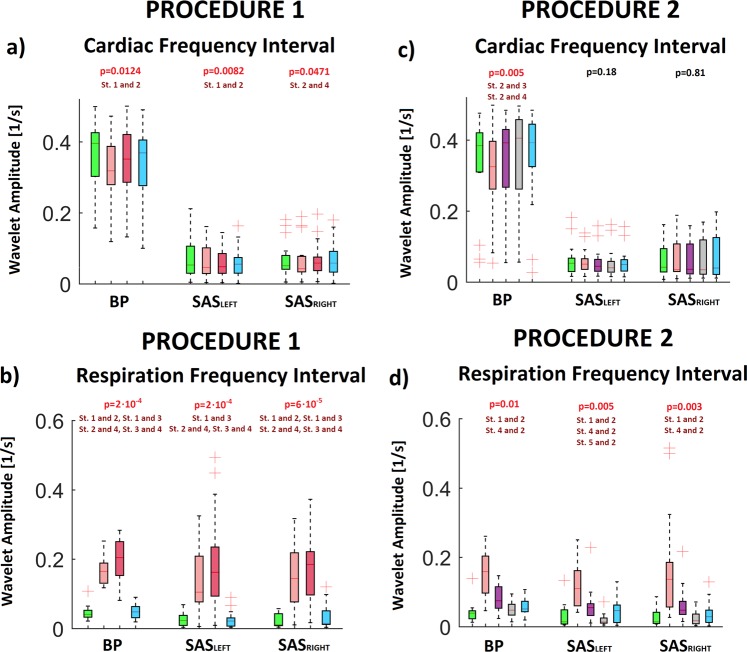
The results of the analysis for all collected signals: BP, SAS_LEFT_ and SAS_RIGHT_. The a and c (b and d) panels correspond to the WT amplitudes from cardiac (respiration) frequency interval. The a and b (c,d) panels correspond to first (second) procedure. The values of “p” was estimated using Friedman test. Post hoc test comparison (Tukey test) was used to find differences between stages of procedure. Symbol “St.1 and 2” means that stages 1 differ significantly from stages 2.

The results of Friedman test for both procedures was displayed in Fig. 4. Excluding SAS signals (both hemispheres) for cardiac frequency interval for procedure 2 the wavelet amplitude for all signals was statistically significant (p < 0.05).

**Figure 5:**
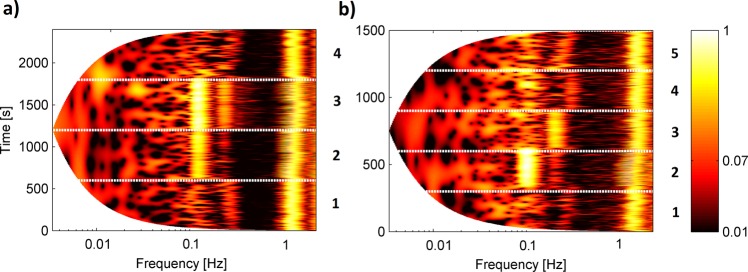
(**a**) Cross Wavelet Power (CWP) of procedure 1 for one of the volunteer. Four numbers correspond to different stages of procedure (see Fig. 1). (**b**) Cross Wavelet Power of procedure 2 for one of the volunteer. Five numbers correspond to different stages of procedure (see Fig. 2). The CWP was estimated for BP and SAS_LEFT_ signals.

Figure 5 illustrates the results of applying the cross wavelet transform (CWP) for 40 minutes of procedure 1 (a) and 25 minutes of procedure 2 (b). The value of CWP was estimated for BP and SAS_LEFT_ signals. It is clearly visible that both signals have a cardiac component with a frequency about 1 Hz and a respiration components: 0.1 Hz, 0.2 Hz and 0.3 Hz during both procedures.

**Figure 6:**
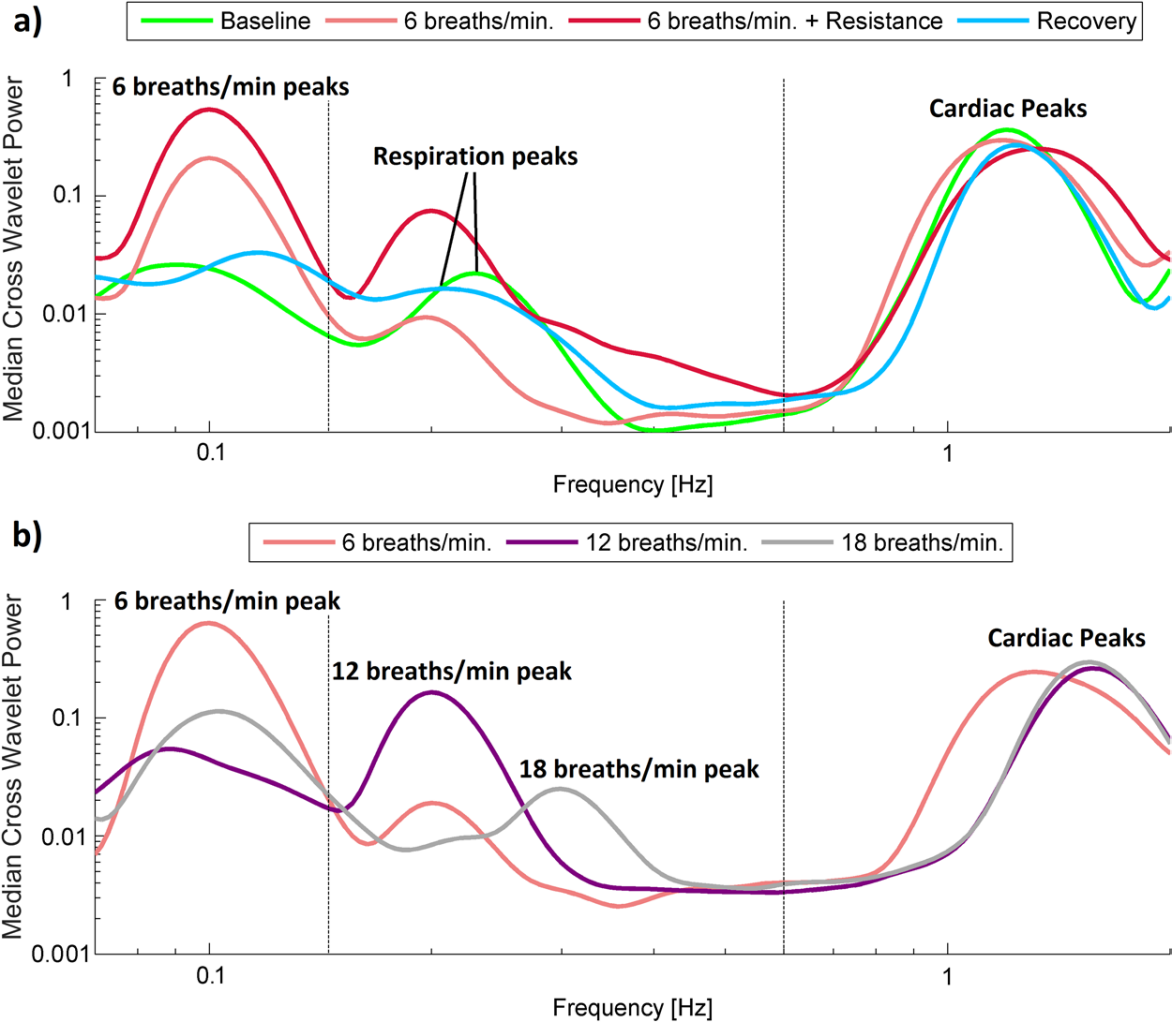
Median of the time-averaged CWP of BP and SAS_LEFT_ signals recorded in all subjects for (**a**) procedure 1 and (**b**) procedure 2. Various line colours correspond to different stages of procedure.

To simplify the comparison of estimated CWP in terms of their frequency content, we plotted Fig. 6, which illustrates the median of the CWP for procedure 1 (a) and procedure 2 (b). The values of the peaks for each volunteer were subjected to statistical analysis to establish more quantitatively the difference between subjects for different stages of the procedures.

**Figure 7:**
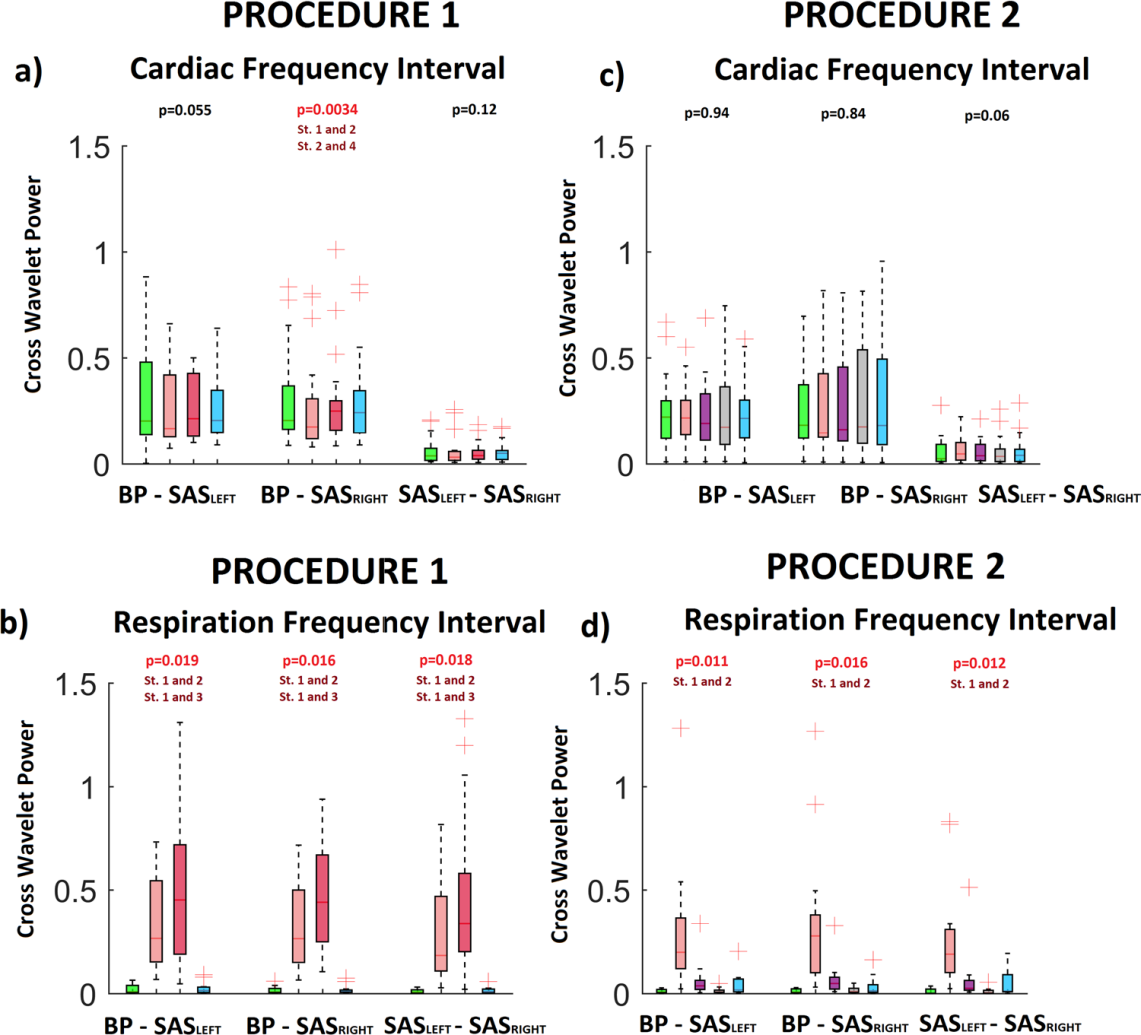
The results of the statistical analysis for CWP for all combinations between collected signals: BP - SAS_LEFT_, BP - SAS_RIGHT_ and SAS_LEFT_ -SAS_RIGHT_. The top (bottom) panels correspond to the CWP peaks from cardiac (respiration) frequency interval. The left (right) panels correspond to first (second) procedure. Various box colours correspond to different stages of procedure (see legend of Fig. 6).

The results of our statistical analysis illustrate Fig. 7. CWP for all combinations between measured signals was statistically significant for respiration frequency interval. For cardiac frequency interval only value of CWP between BP and SAS_RIGHT_ signals was statistically significant.

**Figure 8:**
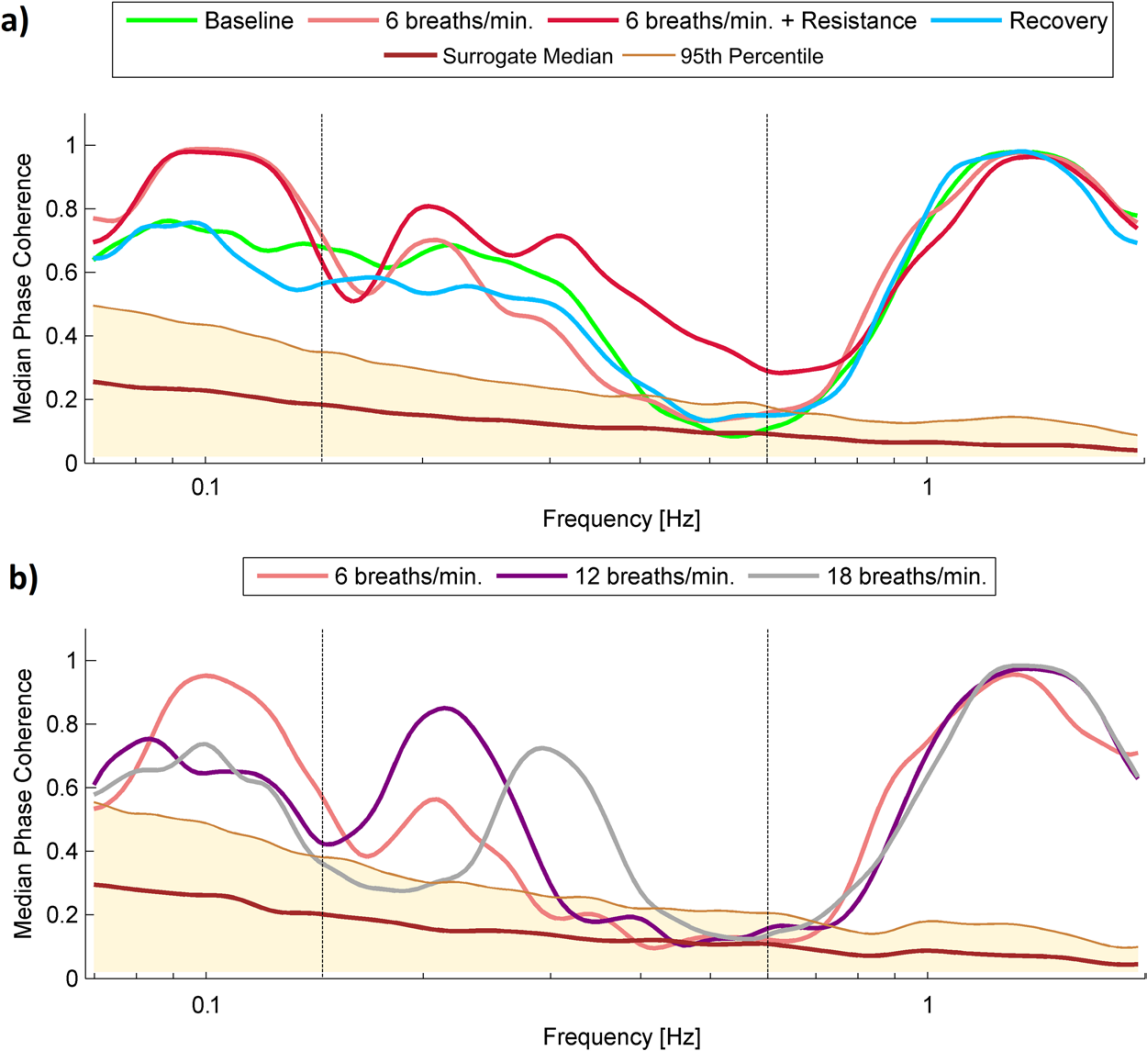
(**a,b**) Median of the time-averaged WPCO of BP and SAS_LEFT_ signals recorded in all subjects for (**a**) procedure 1 and (**b**) procedure 2. Various line colours correspond to different stages of procedure.

Wavelet phase coherence (WPCO) were calculated between BP and both (left and right) SAS signals, and directly between the SAS signals. Figure 8 shows median of WPCO estimated for BP and SAS_LEFT_ signals for procedure 1 (panel a) and procedure 2 (panel b). Phase coherence at each frequency was considered significant if its value was above the 95^th^ percentile of 380 intersubject surrogates. All statistically significant WPCO peaks were subjected to statistical analysis to establish more quantitatively the difference between subjects for different stages of the procedures.

**Figure 9:**
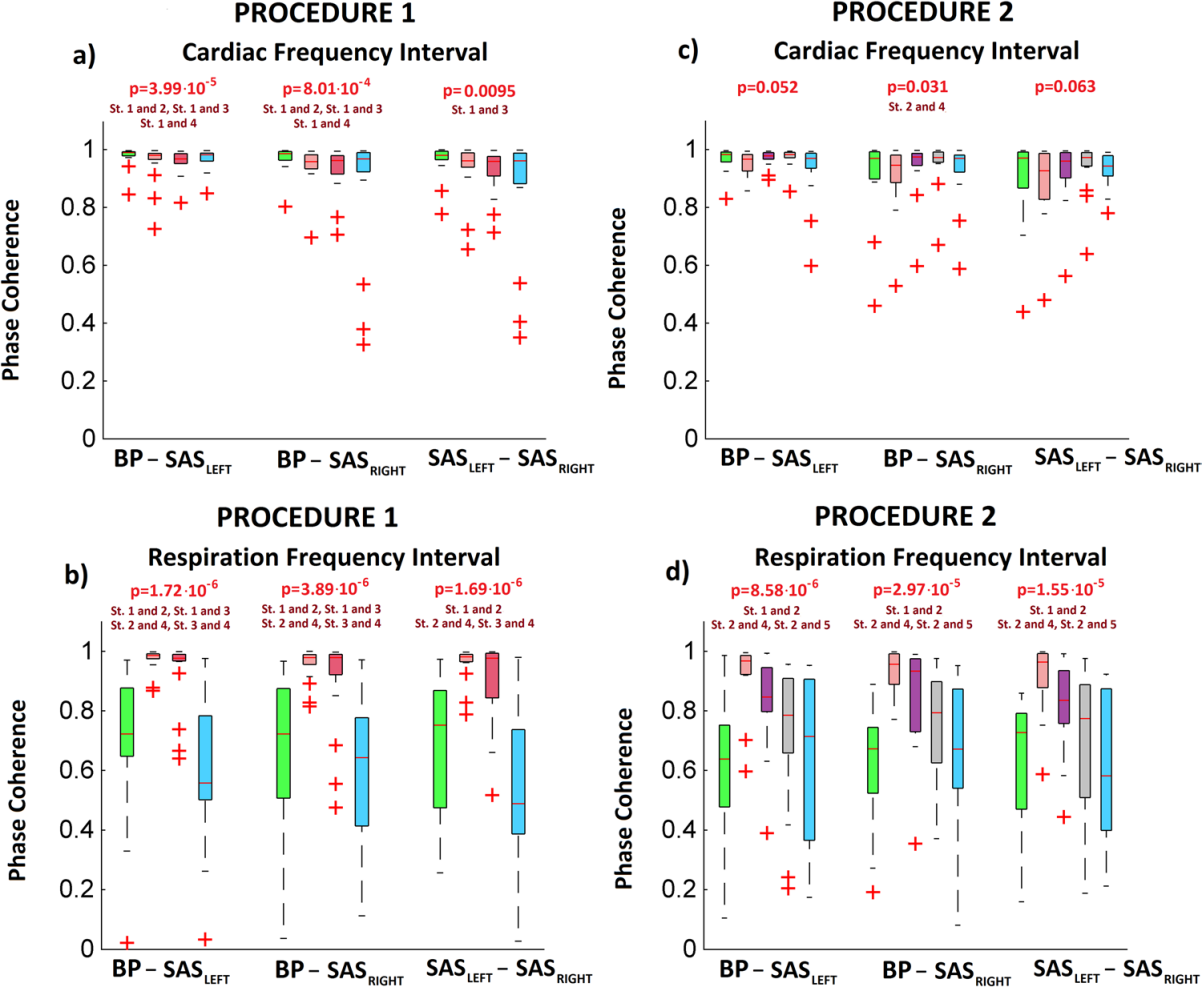
The results of the statistical analysis for WPCO for all combinations between collected signals: BP - SAS_LEFT_, BP - SAS_RIGHT_ and SAS_LEFT_ -SAS_RIGHT_. The top (bottom) panels correspond to the WPCO peaks from cardiac (respiration) frequency interval. The left (right) panels correspond to first (second) procedure. Various box colours correspond to different stages of procedure (see legend of Fig. 7).

The results of our statistical analysis illustrate Fig. 9. WPCO for all combinations between measured signals was statistically significant for cardiac and respiration frequency interval (excluding WPCO for second procedure, cardiac interval, signal combinations: BP-SAS_LEFT_ and SAS_LEFT_- SAS_RIGHT_).

## Discussion

Our study has demonstrated that the overall increases in BP and SAS oscillations observed during slow breathing are strictly dependent on the respiratory component, and this phenomenon is particularly evident when inspiratory resistance is applied (Fig. 4b). Second, drop in cardiac component of BP amplitude evoked by slow-breathing may be perceived as a cardiovascular protective mechanism to avoid target organ damage. Third, slow breathing results in coherence reduction of BP and SAS oscillations (both amplitudes and phases, Figs 7a and 9a, respectively) at cardiac frequency.

The beneficial cardiovascular effects of slow breathing are usually explained by the increase in both baroreceptors’ sensitivity and heart rate variability (reviewed by^1^). We now report an additional mechanism evoked by controlled slow breathing: a lessening of cardiac-derived BP pulsatility. Physiologically, there are two main mechanisms influencing blood flow velocities. One is dependent on vessels’ performance, where arteries’ buffering capacity protects from pressure-related damage to peripheral organs. As documented in human studies, increased arterial stiffness (e.g. commonly seen in long-lasting hypertension or obstructive sleep apnoea) confers cardiovascular risk and translates into higher rates of target organ damage^27,28^. The other regulatory component implicated in blood flow pulsatility is dependent on the heart’s performance, where interaction between HR and SV plays an essential role. The respiration synchronous variation in SV is driven by changes in intrathoracic pressure and direct mechanical interactions between the right and the left ventricles^29–32^.

Exposure to highly pulsatile pressure is a known predictor of cerebral vascular damage and increased risk of cerebrovascular events, even in the absence of elevated MAP^33–35^. Therefore, the observed decline in cardiac-related BP pulsatility may be especially beneficial for cerebral circulation. Cerebrovascular pulsatility plays an important role as it may directly affect brain functioning and structures in the longer perspective. The SAS oscillations at 1 – 1.5 Hz frequency are predominantly dependent on the heart rate^20,36^ and cardiac output^37^. Augmented pulsatile cerebrospinal fluid (CSF) motion has been recently linked to impairments in white matter structure and function in ageing^38^, hypertension^39^ and multiple sclerosis^40^. Thus, controlled slow breathing techniques may offer a non-pharmacological aid to decrease heart-driven CSF oscillations. Importantly, our study suggests that duration of slow breathing exercise matters; a lessening of cardiac-derived BP pulsatility was statistically significant only in procedure lasting 10 minutes. 5 minutes period of slow breathing is too short to evoke described changes. In future studies the optimal time to achieve maximal benefit for the patients should be established.

Increased respiratory resistance set at −7 cm H_2_O elevates both BP and cerebral blood flow velocity oscillations within frequency range (0.04 to 0.4 Hz) during simulated hypovolemia. Interestingly, mean cerebral blood flow velocity is not affected^41^. In this study presyncopal symptoms were delayed by ∼4 min (245 s) when subjects were exposed to enhanced BP and cerebral blood flow velocity oscillations. Consequently, regular respiratory driven CBF fluctuations into higher maximum velocities may represent a protective mechanism for maintaining adequate cerebral perfusion that delays the onset of presyncopal symptoms and prolongs tolerance to hypovolemia^41^. Similarly, observed in our study augmented BP and CSF oscillations at respiratory frequency can potentially reflect similar phenomena (i.e. support brain perfusion). In addition we may speculate that increased CSF motion at respiratory and slower frequencies may support interstitial fluid flow in the brain^42^.

To the best of our knowledge we demonstrated for the first time that slow breathing results in lower coherence of cardiac components of BP and SAS oscillations. We analysed both amplitude (CWP, Fig. 7) and phase coherences (Fig. 9) of both signals, and both declined during slow breathing. Phase difference analysis suggests that at the cardiac frequency both signals are largely independent and oscillations are generated by the heart. Nevertheless, phases of BP and SAS are highly coherent^22^. This mechanism is apparently weakened by slow breathing. As both BP and SAS oscillations are heart driven^22,36^ BP-SAS lower coherence during slow breathing might be related to augmented heart rate variability^1,43^.

HR remained stable only during slow breathing without resistance (Table 1), which is in line with previous reports on the cardiovascular and respiratory effects of yogic slow breathing among yoga beginners^44^. The slow breathing augments the tidal volume and stimulates the Hering-Breuer reflex, an inhibitory reflex triggered by stretch receptors in the lungs that stimulate vagus afferents^45^. However, during resistance slow breathing, the additional effort to inhale augments sympathetic activity^46^ and results in, as observed in our study, HR acceleration.

Inspiratory resistance resulted also in wavelet amplitude coherence of BP-SAS signals recovery (CWP increase, Fig. 7a). Consequently, this study support our earlier finding that BP-SAS amplitude coherence at cardiac frequency tend to be stabilised by sympathetic nervous system^21,47^. Interestingly, BP-SAS phase coherence remained diminished after inspiratory resistance was applied (Fig. 9a). Therefore, we may speculate that phase coherence is less affected by changes in autonomic nervous system activity. Importantly, to restore BP and SAS amplitudes to the values observed in control conditions (Fig. 4a) recovery of wavelet amplitudes coherence (Fig. 7a) was the key element, while diminished phase coherence (Fig. 9a) did not seem to play a role. It may support the concept of BP and SAS signals entrainment.

Interestingly, an increased inspiratory resistance breathing augmented SAS width (Table 1). This phenomenon may be explained by increased SAS amplitude resulting from respiratory motion. During inspiration, due to inspiratory negative pressure in the thoracic cavity, the spinal epidural veins are emptied, resulting in caudal movement of CSF flow in the spinal canal. Furthermore, decreased thoracic pressure affects the hydrostatic pressure that drives the low-resistance paravenous, venous and lymphatic CSF drainage^12,48^. All of these changes are enhanced by deep inspiration^49,50^. Importantly, slow breathing and slow breathing with inspiratory resistance augmented both amplitude (CWS, Fig. 7b) and phase coherence (Fig. 9b). Consequently, even if BP and SAS oscillators seem independent at resting state^22^, the signals become more coherent at respiratory frequency.

Breathing at controlled rate for 20 to 30 minutes (depending on the procedure) is challenging even to healthy subjects. Therefore, we limited the number of measured signals, for instance we did not record a real-time sympathetic firing with microneurography. The volunteers exercised symmetrical breathing which is closer to yoga ujjayi breathing than to physiological breathing (1/3 for inspiration and 2/3 for expiration). However, symmetrical breathing was reported to be the best technique for improving baroreflex sensitivity in yoga-naive subjects while differences between symmetrical and non-symmetrical breathing are very limited^44^. Finally, SAS Monitor does not measure absolute width of the SAS expressed e.g. in millimetres. The NIR-T/BSS signal provides information on SAS changes in time.

Recent studies suggest the long-term beneficial effect of slow breathing might be visible in heart-failure patients^51^ while is not present in healthy subjects^52^. Our experiments were performed in non-trained yoga-naïve subjects. We observed the effects of controlled (timed) breathing imposed by visual signals. Based on available data we are not able to say whether BP and SAS oscillations alter chronically and consequently, whether observed phenomena provide long-term BP-SAS regulation and organ protection. Future studies with pre-trained or yoga-breathing practitioners are warranted to assess long-term effects of slow breathing on BP-SAS oscillations.

The results of our study are highly consistent with previous reports, which definitely stand for reliability of the applied techniques and findings (e.g. the observed acute BP decline during slow breathing^5,8,9^, HR accelerations when inspiratory resistance applied^42^, increase blood oxygenation regardless the application of inspiratory resistance^1^. Additionally, the study participants were able to avoid hyperventilation with subsequent changes in EtCO_2_, which allowed to precisely delineate regulatory mechanisms. All the aforementioned aspects increase reliability of our results.

## Conclusions

Using integrated non-invasive techniques, we have demonstrated for the first time that slow breathing diminishes cardiac-dependent BP and CSF pulsatility. BP and SAS oscillators become less coherent at cardiac frequency during slow breathing. We unveiled a regulatory mechanism evoked by controlled slow breathing which adds to our understanding of the beneficial cardiovascular effects of slow-breathing techniques. Augmented BP and SAS amplitude observed in slow breathing are mainly related to respiration, which is especially seen when inspiratory resistance is applied. BP and SAS oscillators become more coherent at respiratory frequency during slow breathing.

## Materials and Methods

### Subjects

Experiments were performed with a group of 20 healthy volunteers (8 males and 12 females, age 25.3 ± 7.9 years, BMI = 22.1 ± 3.2 kg/m^2^). None of them were smokers or received any chronic pharmacotherapy (including diet supplements), nor were any of the participants involved in advanced yoga breathing or meditation techniques such as resistance breathing. This study was carried out in accordance with the recommendations of Helsinki. The experimental protocol and the study were approved by the Ethics Committee of Medical University of Gdansk (NKBBN/265/2016). All subjects were informed in detail about the study’s objectives and any potential hazard to their health. All volunteers gave written informed consent to participate in the study. Participants were asked to refrain from coffee, tea, cocoa and any food and beverages containing methylxanthine for at least 8 hours before the tests. All procedures were preceded by 10 minutes of rest in the sitting position in a comfortable and quiet room.

### Experimental design

All tests were conducted in a quiet room with a comfortable temperature. Two procedures, as described below, were performed in the morning on two consecutive days. First, subjects were asked to breathe with non-rebreathing low pressure, low resistance, low death space sealed face mask. Each study participant spent approximately 20 minutes learning how to breathe wearing the study equipment prior to commencement of the designated procedures. Participants were advised to avoid hyperventilation (very deep inhalations). Following the mask fixation and dry-run practicing with the equipment, subjects were asked to rest before the baseline recordings were initiated. Inspiration and expiration pace was imposed by visual signals displayed on the computer screen.

Procedure 1 consisted of spontaneous breathing and of breathing at controlled rates of 6 breaths/minute followed by 6 breaths/minute with inspiratory resistance (10-minute intervals; see Fig. 1). Increased inspiratory resistance was controlled with a modified single-use Threshold IMT® device (Phillips-Respironics, Best, the Netherlands), designed for rehabilitation of the inspiratory muscles. The device consisted of a mouthpiece, a container and a calibrated adjustable valve which allowed for precise control of the inspiratory resistance, ranging from −2 to −40 cm H_2_O. The target pressure of the generated inspiratory resistance equalled −20 cm H_2_O. Procedure 2 consisted of breathing spontaneously and at controlled rates of 6, 12 and 18 breaths/minute for 5 minutes (Fig. 2).

The participants exercised controlled slow breathing in a symmetrical pattern, that is, both inspiration and expiration were timed and lasted 5 seconds each. During breathing at 12 breaths per minute, both inspiration and expiration lasted 2.5 seconds. Finally, during fast breathing (18 breaths per minute), both inspiration and expiration lasted 1.67 seconds.

### Measurements

BP and heart rate (HR) were measured using continuous finger-pulse photoplethysmography (CNAP, CNSystems Medizintechnik AG, Graz, Austria). Finger BP was calibrated against brachial arterial pressure. SAS (SAS_LEFT_ – left hemisphere, SAS_RIGHT_ – right hemisphere) signals were recorded using SAS Monitor (NIRTI SA, Wierzbice, Poland). Detailed description of SAS Monitor was provided previously^22^. Oxyhaemoglobin saturation (SaO_2_) was measured using a medical monitoring system (Datex-Ohmeda, GE Healthcare, Wauwatosa, WI, US). Gas samples from the mouthpiece were constantly analysed using the side-stream technique for end-tidal CO_2_ (EtCO_2_) with the metabolic module of the same medical monitoring system. All parameters were recorded continuously for further analysis, and BP and SAS signals were synchronized on a beat-to-beat basis^22^.

### Wavelet transform

To detect physiological processes that are responsible for generating oscillations in the cardiovascular system, we need an appropriate mathematical tool. One such tool is the wavelet analysis with the Morlet mother wavelet, which can detect these oscillations with logarithmic frequency resolution and follow the variation of their frequencies and amplitudes in time^25^. The wavelet transform is a method that transforms a signal from the time domain to the time-frequency domain. The definition of the wavelet transform is:

$$W(s,t)=\frac{1}{\sqrt{s}}{\int }_{-\infty }^{+\infty }\phi (\frac{u-t}{s})g(u)du,$$

where *W*(*s*,*t*) is the wavelet coefficient, *g*(*u*) is the time series and *φ* is the Morlet mother wavelet, scaled by factor *s* and translated in time by *t*. The Morlet mother wavelet is defined by the equation:

$$\phi (u)=\frac{1}{\sqrt[4]{\pi }}\exp (-i2\pi u)\exp (-0.5{u}^{2}),$$

where $i=\sqrt{-1}$. The reason for using the Morlet wavelet is its good localization of events in time and frequency due to its Gaussian shape^26,53^. The wavelet coefficients are complex numbers in the time-frequency plane when the Morlet wavelet is used:

$$X({\omega }_{k},{t}_{n})={X}_{k,n}={a}_{k,n}+i{b}_{k,n}.$$

They define the instantaneous relative phase,

$${\theta }_{k,n}=\arctan (\frac{{b}_{k,n}}{{a}_{k,n}}),$$

and the absolute amplitude,

$$|{X}_{k,n}|=\sqrt{{a}_{k,n}^{2}+{b}_{k,n}^{2}},$$

for each frequency and time.

During the measurement, external factors such as respiration or heartbeat may create an amplitude and phase modulations. Mathematical tool to find relationship between an amplitude of two signals is the cross wavelet transform defined as^54^:

$${C}_{A}({f}_{k},{t}_{n})={X}_{1}{({f}_{k},{t}_{n})}^{\ast }{X}_{2}({f}_{k},{t}_{n}),$$

where * denotes complex conjugation. To analyse our data we used the cross wavelet power $\,|{C}_{A}({f}_{k},{t}_{n})|$ (CWP). Another useful tool which enables us to determine whether the oscillations detected are significantly correlated over time is the wavelet phase coherence (WPCO). To estimate the WPCO first we must calculate instantaneous phases at each time *t*_*n*_ and frequency *f*_*k*_ $({\theta }_{k,n}=\arctan (\frac{{b}_{k,n}}{{a}_{k,n}}))$ for both signals and next calculate the amplitude of time average using the following expression^55,56^:

$${C}_{\theta }({f}_{k})=\frac{1}{n}|\sum _{t=1}^{n}\exp [i({\theta }_{2k,n}-{\theta }_{1k,n})]|.$$

The value of the WPCO function *C*_*θ*_(*f*_*k*_) is between 0 and 1. When two oscillations are unrelated, their phase difference continuously changes with time, thus their *C*_*θ*_(*f*_*k*_) approaches zero. If the *C*_*θ*_(*f*_*k*_) is around 1, the two oscillations are related and the phase difference between the two signals at a particular frequency remain constant.

### Surrogate Test Based on Intersubject Signal Pairs

Surrogate data testing is a method which is used to test whether the estimated values of phase coherence are statistically significant or not. The method has been successfully used in the analysis of signals from living systems^57,58^.

As we know for oscillations with lower frequencies, there are fewer cycles within a given time interval. This causes artificially increased phases coherence, even in cases where there is none. We must find a significance level above which the phase coherence may be regarded as physically meaningful. To estimate significance levels for the wavelet phase coherence, we used intersubject surrogates^54^. This assumes that the signals collected from different subjects must be independent, while having similar characteristic properties. We therefore calculated surrogate values of, for example, the BP phase coherence using a signal from one subject and the SAS from another. In our studies, we analysed data from 20 subjects. The significance level was estimated as the 95^th^ percentile of 380 (2-permutations of 20 subjects) intersubject surrogates. The actual value of phase coherence obtained at each frequency can then be compared with the surrogate threshold. When the phase coherence is located above the threshold it is considered to be statistically significant.

### Statistical analysis

Nonparametric statistical tests were used for all comparisons, to avoid the assumption of normality in the results. A one way, between stages of both procedures, non-parametric ANOVA (Friedman test) was conducted to find the differences among stages in the procedures. Post hoc comparisons, using Tukey test, was used to find differences between stages of procedure when Friedman test was statistically significant (p < 0.05). The results of our calculations we placed in Figs 4, 7, 9 and Tables 1, 2.

## Data Availability

All data generated or analysed during this study are included in this published article.
